# Reliability of Cancer Treatment Information on the Internet: Observational Study

**DOI:** 10.2196/10031

**Published:** 2018-12-17

**Authors:** Ryo Ogasawara, Noriyuki Katsumata, Tatsushi Toyooka, Yuko Akaishi, Takaaki Yokoyama, Gemmu Kadokura

**Affiliations:** 1 Nippon Medical School Tokyo Japan; 2 Department of Medical Oncology Nippon Medical School Musashikosugi Hospital Kawasaki City Japan

**Keywords:** internet, cancer treatment, clinical practice guideline, mobile phone

## Abstract

**Background:**

Finding the correct medical information in a flood of information from the internet is a significant issue for patients with cancer.

**Objective:**

We investigated the reliability of the information on cancer treatment methods available on the internet based on an evaluation by medical oncologists, medical students, and cancer survivors.

**Methods:**

Using Google and Yahoo as the search engines, we carried out the information search using 2 keywords, “cancer treatment” and “cancer cure,” and the top 20 information sites were identified. A similar search was conducted on 5 types of cancer. The reliability of the information presented was rated on a 3-level scale (A, B, or C). Level A referred to reliable sites (providing information complying with the clinical practice guidelines for various types of cancer), Level B included sites not falling under either Level A or Level C, and Level C comprised dangerous or harmful sites (providing information on treatment not approved by the regulatory authority in Japan and bombastic advertisements without any relevant clinical evidence). The evaluation was conducted by medical oncologists, medical students, and cancer survivors. The consistency of the information reliability level rating between the medical students or cancer survivors with that of the medical oncologists was assessed by using the kappa value.

**Results:**

A total of 247 sites were evaluated for reliability. The ratings provided by the medical students’ group were as follows: Level A, 12.1% (30/247); Level B, 56.3% (139/247); and Level C, 31.6% (78/247). The ratings provided by the cancer survivors’ group were as follows: Level A, 16.8% (41/244); Level B, 44.7% (109/244); and Level C, 38.5% (94/244). The ratings provided by the oncologists’ group were as follows: Level A, 10.1% (25/247); Level B, 51.4% (127/247); and Level C, 38.5% (95/247). The intergroup rating consistency between the medical students’ group and oncologists’ group was 87.4% (216/247, kappa=0.77) and that between the cancer survivors’ group and oncologists’ group was 76.2% (186/244, kappa=0.61).

**Conclusions:**

Of the investigated sites providing information on cancer treatment on the internet, the percentage of sites that seemed to provide harmful information was much higher than that of sites providing reliable information. The reliability level rating was highly consistent between the medical students’ group and the medical oncologists’ group and also between the cancer survivors’ group and the medical oncologists’ group.

## Introduction

With the recent advances in cancer treatment, the therapeutic options (surgical treatment, drug therapy, radiotherapy, etc) are expanding and becoming more complicated. Therefore, for patients with cancer, selection of the appropriate treatment options is a significant issue.

Cancer patients seek information about cancer diagnosis, diagnostic tests, treatment options, complications, prognosis, etc, and they often search for information by themselves on the internet. The percentage of people with access to the internet now exceeds 80%, and the number of internet users has continued to increase year after year [1]. However, in Japan, according to one report, the probability of internet users finding correct information on the internet using search engines such as Google Japan and Yahoo Japan does not exceed 50%, and 10% of the information accessed by the search are advertisements [2]. Thus, sufficient information on the methods available for cancer treatment is not available on the internet or in publications that are easily accessible by cancer patients. Furthermore, many of the treatment methods described in websites on the internet are not reliably effective, and advertisements overemphasizing their efficacy are often found. It is not uncommon for unapproved treatments without any evidence of efficacy (eg, high dose vitamin C therapy, some kinds of immune cell therapy) to be provided at various private clinics as a treatment not covered by health insurance, necessitating high out-of-pocket payments by the patients. Incorrect information is found in abundance on the internet, which can cause misunderstanding and erroneous knowledge in patients.

In Japan, it is difficult for cancer patients to select the correct information from the internet. There is also a report suggesting that the health literacy of the Japanese population is lower than that of Europeans [3]. Therefore, we conducted this study to investigate the current status and reliability of the information on cancer treatment available on the internet, with the goal of devising appropriate educational campaigns on standard cancer treatments in Japan.

## Methods

### Recruitment

The internet search engines Google and Yahoo were employed to collect information from the internet. The search was conducted using the 2 keywords “cancer treatment” and “cancer, cure” (both in Japanese expressions), and the top 20 sites providing the information needed were identified. A similar search was also done on each of 5 major types of cancer (lung cancer, breast cancer, stomach cancer, colorectal cancer, and liver cancer).

All the information obtained was evaluated for reliability according to 2 criteria: (1) The source of information is described and the sources are based on reliable cancer practice guidelines (Japan Society of Clinical Oncology; Japanese Society of Medical Oncology; Japanese Society for Palliative Medicine; National Cancer Center for Cancer Control and Information Services, Japan; Cancer Information Japan, Japanese version of the Perceived Deficits Questionnaire; Japanese version of the National Comprehensive Cancer Network Guidelines; the Medical Information Network Distribution Service Guideline Center; etc) and (2) The information is not approved by the regulatory authority in Japan, markedly deviates from cancer practice guidelines, includes bombastic advertisements without any relevant clinical evidence, and can be considered as being potentially harmful to patients (eg, sites guiding patients to medical facilities or the like that provide treatments that are not approved, are not acknowledged as standard therapy, or are not designated as frontier therapy by the government and sites having links to food supplement marketing or advertisement pages, etc)

Using the aforementioned criteria, the reliability of the information was rated on a 3-level scale as follows: Level A: reliable sites, satisfying criterion (1) and not apparently falling under (2); Level B: falling under neither Level A nor C; and Level C: dangerous or harmful sites, not satisfying (1) and evidently falling under (2), and unclassified sites that do not describe any treatment method.

The evaluation was conducted by a medical students’ group (3 medical students: RO, TT, and YA) and a cancer survivors’ group (3 cancer survivors: Kimiko Ohi, Yumi Higure, and Yukari Tanaka). The cancer survivors provided consent for participating in this study as volunteers (2 women aged between 50 and 59 years and 1 woman aged between 60 and 69 years; 2 were university graduates, and 1 was a junior college graduate). Before performing the evaluation, each member of the group received a 30-minute lecture from a medical oncologist (NK) about the evaluation method. If all 3 members of the group gave the same rating, that rating was adopted as the reliability level for the site concerned. If the rating differed among the members, the reliability level of the site was finally decided through discussion among the members. There were 3 medical oncologists (2 board-certified medical oncologists and 1 not certified) who also individually rated the reliability level of each site. If the rating differed among the oncologists, the rating to be finally adopted was decided through discussion among the oncologists.

### Statistical Analysis

Data were analyzed using IBM SPSS Statistics version 20 (IBM Corp, Armonk, NY, US). The categorical data for each keyword was analyzed by the chi-square test and the Friedman test. The consistency of rating between each of the cancer survivors’ and medical students’ group and the medical oncologists’ group was evaluated through calculation of the kappa value. The consistency of rating between any 2 groups was analyzed by determining the Cohen kappa coefficient and that among the 3 groups was analyzed by determining the Fleiss kappa coefficient [4]. Interpretations of the kappa statistic were based on the criteria described by Landis and Koch [5], that is, the level of reliability was defined as follows: kappa values of 0.81-1.00, near-perfect or perfect agreement; 0.61-0.80, substantial agreement; 0.41-0.60, moderate agreement; 0.21-0.40, fair agreement; and 0.01-0.20, slight agreement.

## Results

The top 20 sites hit by the search using each engines (Google Japan and Yahoo Japan) were evaluated after the elimination of duplications. The search was conducted on June 15, 2016. Among the 480 sites accessed, the top 20 sites hit by the search using the keywords “cancer, cure,” “lung cancer treatment,” “lung cancer,” “breast cancer treatment,” “breast cancer cure,” “stomach cancer treatment,” “stomach cancer, cure,” “colorectal cancer treatment,” “liver cancer treatment” and “liver cancer, cure” were completely consistent between Google and Yahoo, and the sites hit by the search conducted using the keywords “cancer treatment” and “colorectal cancer, cure” were partially consistent between Google and Yahoo. These sites were counted as duplications and excluded from evaluation. When the search was made using the keyword “breast cancer treatment,” a link to 1 of the 20 top sites was lost during the evaluation, and this site was excluded from the analysis, with the remaining 19 sites included in the analysis. There were 3 sites hit by the Yahoo search using the keywords “colorectal cancer, cure” that were not evaluated by the cancer survivors’ group. In total, 247 sites were evaluated by both the oncologists’ group and medical students’ group, and 244 sites were evaluated by the cancer survivors’ group as the top 20 sites yielded by the Google and Yahoo searches using the aforementioned keywords.

**Figure 1:**
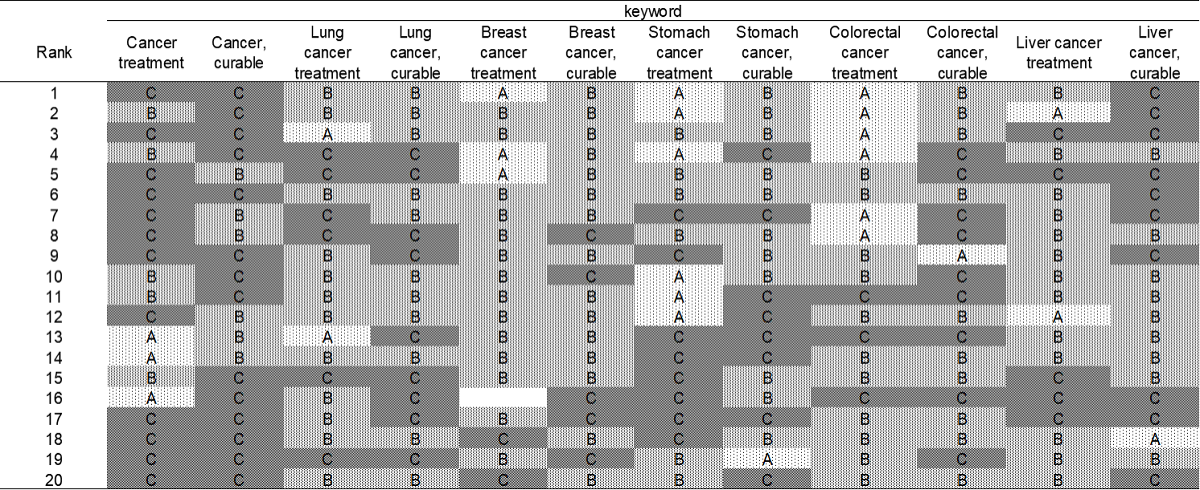
Evaluation by medical oncologists' group of the top 20 sites hit by Google search (June 15, 2016).

**Figure 2:**
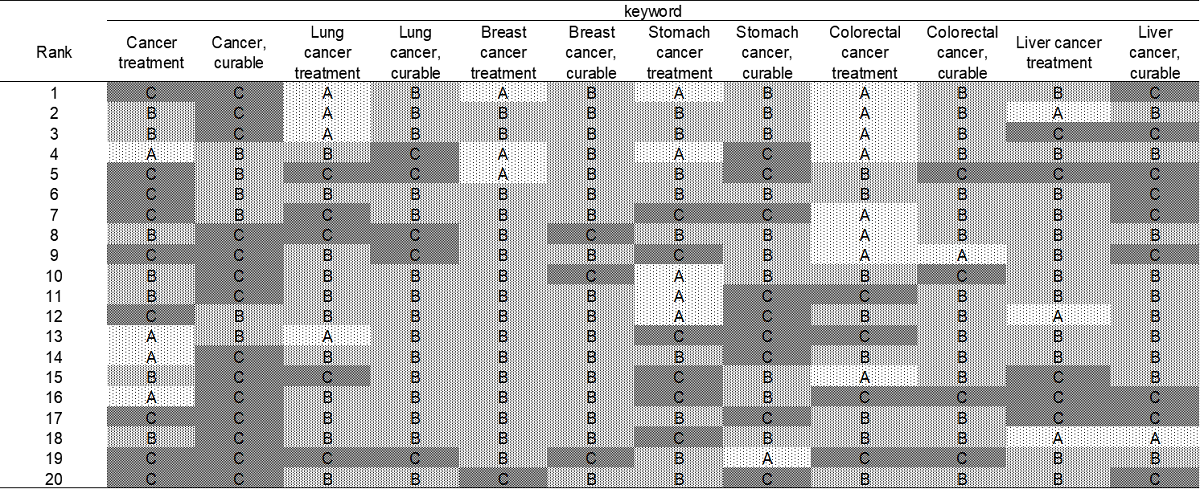
Evaluation by a medical students' group of the top 20 sites hit by Google search (June 15, 2016).

**Figure 3:**
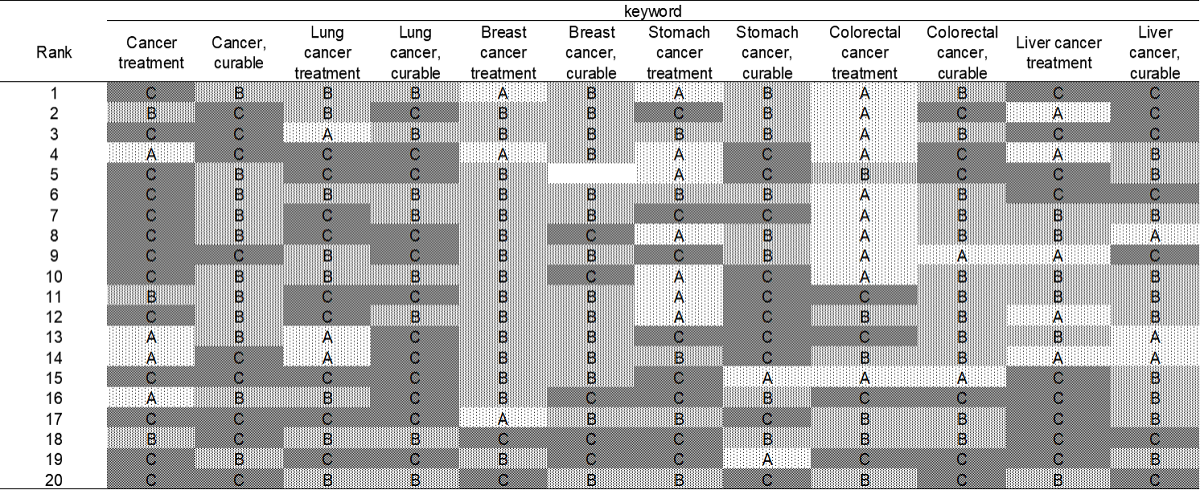
Evaluation by cancer survivors' group of the top 20 sites hit by Google search (June 15, 2016).

Out of the 247 sites, the oncologists’ group provided a Level A rating for 25 sites (10.1%), Level B rating for 127 sites (51.4%), and Level C rating for 95 sites (38.5%; Figure 1); the medical students’ group gave a Level A rating for 30 sites (12.1%), Level B rating for 139 sites (56.3%), and Level C for 78 sites (31.6%; Figure 2); and the cancer survivors’ group provided a Level A rating for 41 sites (16.8%), Level B rating for 109 sites (44.7%), and Level C rating for 94 sites (38.5%; Figure 3). The number of sites rated as Level A was the smallest for the oncologists’ group, differing significantly from that for the cancer survivors’ group (oncologists’ group vs medical students’ group: *P*=.47, oncologists’ group vs cancer survivors’ group: *P*=.03, medical students’ group vs cancer survivors’ group: *P*=.16).

Of the 124 sites hit by the search using the keyword “treatment,” 22 sites (17.7%) were rated as Level A, 62 sites (50.0%) as Level B, and 40 sites (32.3%) as Level C. Of the 123 sites yielded using the keyword “cure,” 3 sites (2.4%) were rated as Level A, 65 sites (52.8%) as Level B, and 55 sites (44.7%) as Level C. The number of sites with Level A rating was higher among the sites hit using the keyword “treatment,” than among the sites hit using the keyword “cure” (*P*<.001).

According to cancer type, the rating by the oncologists’ group for the 20 sites hit using the keyword “lung cancer treatment” was Level A for 2 sites (10%), Level B for 12 sites (60%), and Level C for 6 sites (30%). The ratings for the sites by the oncologists’ group were as follows: among the 20 sites hit using the keyword “lung cancer, cure,” Level A for 0 sites (0%), Level B for 11 sites (55%), and Level C for 9 sites (45%); among the 19 sites hit using the keyword “breast cancer treatment,” Level A for 3 sites (16%), Level B for 14 sites (74%), and Level C for 2 sites (11%); among the 20 sites hit using the keyword “breast cancer, cure,” Level A for 0 sites (0%), Level B for 15 sites (75%), and Level C for 5 sites (25%); among the 20 sites hit using the keyword “stomach cancer treatment,” Level A for 6 sites (30%), Level B for 6 sites (30%), and Level C for 8 sites (40%); among the 20 sites hit using the keyword “stomach cancer, cure,” Level A for 1 site (5%), Level B for 11 sites (55%), and Level C for 8 sites (40%); among the 20 sites hit using the keyword “colorectal cancer treatment,” Level A for 6 sites (30%), Level B for 11 sites (55%), and Level C for 3 sites (15%); among the 20 sites hit using the keyword “colorectal cancer, cure,” Level A for 1 site (5%), Level B for 10 sites (50%), and Level C for 9 sites (45%); among the 20 sites hit using the keyword “liver cancer treatment,” Level A for 2 sites (10%), Level B for 13 sites (65%), and Level C for 5 sites (25%); among the 20 sites hit using the keyword “liver cancer, cure,” Level A for 1 site (5%), Level B for 9 sites (45%), and Level C for 10 sites (50%). The number of sites rated as Level A was larger among the sites yielded using the keyword “treatment” than among the sites yielded using the keyword “cure” (lung cancer: *P*=.15, breast cancer: *P*=.06, stomach cancer: *P*=.04, colorectal cancer: *P*=.04, and liver cancer: *P*=.55).

The Friedman test found no significant difference for each keyword among the 3 groups except the keyword “lung cancer, curable,” *P*=.005, and “liver cancer, curable,” *P*=.03.

The number of sites for which the rating was consistent among all 3 members of each group was analyzed. Among the analyzed sites, the ratings were consistent among all 3 members of the cancer survivors’ group for 155 sites (155/244, 63.5%; Fleiss kappa for 3 raters=0.61, 95% CI 0.56-0.66), among all 3 members of the medical students’ group for 201/247 sites (81.4%; Fleiss kappa for 3 raters=0.78, 95% CI 0.72-0.83), and among all 3 members of the oncologists’ group for 232 sites (232/247, 93.9%; Fleiss kappa for 3 raters=0.92, 95% CI 0.87-0.98; Table 1).

If the rating differed among the members of a group, the reliability level of the site concerned was finally decided through discussion among the members. Among the 247 sites (244 sites for the cancer survivors’ group), the number of sites whose reliability level finally adopted through discussion was consistent with the rating by the oncologists group was 186 (186/244, 76.2%) for the cancer survivors’ group (Cohen kappa unweighted=0.61, 95% CI 0.51-0.69) and 216 (216/247, 87.4%) for the medical students’ group (Cohen kappa unweighted=0.77, 95% CI 0.70-0.84; Table 2).

**Table 1:** Consistency of rating among persons within each group.

Search engine and keyword	Frequency of consistency			Total number of sites, n
	Cancer survivors	Medical students	Medical oncologists	
google “cancer treatment”	10	13	17	20
yahoo “cancer treatment”	2	5	5	5
google “cancer, curable”	6	16	17	20
google “lung cancer treatment”	11	17	18	20
google “lung cancer, curable”	11	20	20	20
google “breast cancer treatment”	15	13	19	20
google “breast cancer, curable”	14	20	19	19
google “stomach cancer treatment”	18	17	18	20
google “stomach cancer, curable”	15	18	19	20
google “colorectal cancer treatment”	13	15	19	20
google “colorectal cancer, curable”	14	18	20	20
google “liver cancer treatment”	14	11	19	20
google “liver cancer, curable”	12	15	19	20
yahoo “colorectal cancer, curable”	N/A^a^	3	3	3
Kappa	0.6145	0.7813	0.9296	N/A

^a^N/A: not applicable.

**Table 2:** Consistency of rating by medical oncologists.

Search engine and keyword	Cancer survivors, n	Medical students, n	Total number of sites, n
google “cancer treatment”	17	16	20
yahoo “cancer treatment”	5	5	5
google “cancer, curable”	13	16	20
google “lung cancer treatment”	16	17	20
google “lung cancer, curable”	17	17	20
google “breast cancer treatment”	18	18	20
google “breast cancer, curable”	17	18	19
google “stomach cancer treatment”	14	17	20
google “stomach cancer, curable”	17	19	20
google “colorectal cancer treatment”	15	17	20
google “colorectal cancer, curable”	12	15	20
google “liver cancer treatment”	13	19	20
google “liver cancer, curable”	12	19	20
yahoo “colorectal cancer, curable”	N/A^a^	3	3
Kappa	0.6063	0.7747	N/A

^a^N/A: not applicable.

## Discussion

### Principal Findings

Cancer is an intractable disease and is often incurable. In the present age, the widespread use of smartphones allows easy access to information on the internet, and 60% of Japan obtains health information from the internet [6]. When patients desire information about health, they more often check the internet fist than ask their doctors [7]. Cancer patients also browse the internet often to collect information about cancer [8]. A similar tendency is seen across the world [7]. However, the information available on the internet is often harmful to patients, and there is a report that more than 10% of sites on the internet offering information on lung cancer in Japan recommend alternative therapies [2].

In this study, the number of sites given a reliability rating of Level B or C was larger than the number of sites given a rating of A in each of the evaluations made by the oncologists’ group, medical students’ group, and cancer survivors’ group. This indicates that information on treatment methods based on the relevant guidelines is difficult to obtain from the internet and that the reliability level of the available information on cancer treatment methods on the internet is low in Japan. A report from the United States also shows that there are many sites offering unreliable information on the internet and includes a statement that about half (50%) of the drugs introduced with exaggerative phrases such as “miracle” or “cure” in Google News related to anticancer drugs that were not approved by the Food and Drug Administration, with the patients risking being guided toward adopting treatments of unproven reliability [9]. In this study, however, the prior 30-minute lecture on the evaluation method provided by a medical oncologist resulted in a high consistency of the rating between the cancer survivors’ group and the oncologists’ group (kappa=0.61) and between the medical students’ group and the oncologists’ group (kappa=0.77), although the consistency between the cancer survivors’ group and oncologists’ group was slightly lower than that between the medical students’ group and the oncologists’ group.

### Limitations

This study had several limitations. A 5-level scale of evidence is widely used for critical appraisal for medical information [10]. The validity of the 3-level scale employed in this study remains to be established. The percentage of sites given a reliability rating of Level A was low (10%) in this study, probably because the criteria for Level A adopted in the site information rating step were slightly stringent (requiring guideline-based information and specification of the information source). According to the study reported by Goto et al, about 40% of the sites yielded by Google and Yahoo searches using the keyword “lung cancer” were accorded the highest rating of “acceptable” when a 3-level scale was employed [2]. In addition, the review process has a bias because it is judged by a limited number of each evaluation group. The 2 groups of medical school students and cancer survivors could have some background information about cancer and treatment and receiving the lecture for the evaluation method before scoring websites could have introduced bias. Moreover, more diversified medical experts will be needed for judging the collected data. Furthermore, since the information available on the internet continues to change, and the sites hit as leading sites vary from day to day, extrapolation of the findings from this study to other situations, in general, would probably be unreasonable.

In Japan, physicians can provide health care services not covered by health insurance (ie, services that would require full payment by the patients themselves). Therefore, information on numerous treatment methods, with an emphasis on cancer treatment, is available on the internet. Factors possibly serving as the background for such a situation include: (1) cancer treatment based on guidelines has not spread widely in Japan (as reflected by the small number of sites given a rating of Level A), and (2) under such circumstances, patients with cancer are likely to attempt treatment whose efficacy has not been established if even a slight possibility of cure is promised so that patients can have accurate knowledge about established treatment methods and can be discouraged from seeking unreliable treatments, it may be important to organize educational campaigns across the country and enable cancer patients to select appropriate information from the vast amount of information available on the internet.

This study was designed to evaluate the capability of medical students and cancer survivors to correctly evaluate the information available on the internet. After the medical students and cancer survivors received a lecture to make them aware that cancer treatment based on guidelines on cancer management is the most desirable, they provided ratings that were highly consistent with the ratings provided by the oncologists. This result indicates the importance of dissemination of the information contained in cancer management guidelines among cancer survivors as well as of educational campaigns for the society.

### Conclusions

Although the reliability level of the information on cancer treatment available on the internet seems to be low in general, the results of the 3-level evaluation method employed in this study suggest that judgment of the reliability of individual internet sites can be made relatively easily, even by individuals with poor medical knowledge.
